# Bioactive Metabolites Produced by Cyanobacteria for Growth Adaptation and Their Pharmacological Properties

**DOI:** 10.3390/biology10101061

**Published:** 2021-10-18

**Authors:** Pavitra Nandagopal, Anthony Nyangson Steven, Liong-Wai Chan, Zaidah Rahmat, Haryati Jamaluddin, Nur Izzati Mohd Noh

**Affiliations:** 1 Department of Biosciences, Faculty of Science, Universiti Teknologi Malaysia, Skudai 81310, Malaysia; pavitra1995@graduate.utm.my (P.N.); chanliongwai@graduate.utm.my (L.-W.C.); zaidahrahmat@utm.my (Z.R.); haryatijamaluddin@utm.my (H.J.); 2 Department of Chemistry, Faculty of Science, Universiti Teknologi Malaysia, Skudai 81310, Malaysia; anthony@utm.my; 3 Institute of Bioproduct Development, Universiti Teknologi Malaysia, Skudai 81310, Malaysia

**Keywords:** cyanobacteria, habitat, adaptation strategies, bioactive metabolites

## Abstract

**Simple Summary:**

Cyanobacteria are known as oxygenic microorganisms are able to release oxygen as a byproduct during photosynthesis. Rapidly changing environmental conditions require cyanobacteria to have dynamic adaptation strategies. They synthesize bioactive metabolites that are responsible for protection against harmful environmental conditions and to colonize in various habitats. This review focuses on the roles of bioactive metabolites for cyanobacterial survival and also discusses the bioactivities of these compounds for the treatment of numerous diseases.

**Abstract:**

Cyanobacteria are the most abundant oxygenic photosynthetic organisms inhabiting various ecosystems on earth. As with all other photosynthetic organisms, cyanobacteria release oxygen as a byproduct during photosynthesis. In fact, some cyanobacterial species are involved in the global nitrogen cycles by fixing atmospheric nitrogen. Environmental factors influence the dynamic, physiological characteristics, and metabolic profiles of cyanobacteria, which results in their great adaptation ability to survive in diverse ecosystems. The evolution of these primitive bacteria resulted from the unique settings of photosynthetic machineries and the production of bioactive compounds. Specifically, bioactive compounds play roles as regulators to provide protection against extrinsic factors and act as intracellular signaling molecules to promote colonization. In addition to the roles of bioactive metabolites as indole alkaloids, terpenoids, mycosporine-like amino acids, non-ribosomal peptides, polyketides, ribosomal peptides, phenolic acid, flavonoids, vitamins, and antimetabolites for cyanobacterial survival in numerous habitats, which is the focus of this review, the bioactivities of these compounds for the treatment of various diseases are also discussed.

## 1. Introduction

Cyanobacteria are photosynthetic microorganisms that possess various cellular strategies and physiological capacities to facilitate their adaptations for colonization in diverse environments on Earth. As a result, these photosynthetic microbes can exist in marine, terrestrial, and freshwater habitats. Furthermore, cyanobacteria are the most versatile ancient microorganisms that can thrive in extreme environments such as deserts, polar environments, geothermal springs, hypersaline lakes, and soils with high metal concentrations. They can be classified according to their ability to grow in high pH (alkaliphiles), beneath rock (endolithics), in high salinity (halophiles), under low nutrients (oligotrophics), in low (psychrophiles) or high (thermophiles) temperatures, and under high radiation levels (radiophiles) (Table 1).

Through centuries of evolution, cyanobacteria developed various sophisticated molecular, physiological, and metabolic characteristics for thriving in their habitats. The main aim of this paper is to review the roles of bioactive metabolites in addition to molecular machineries and physiological characteristics in ensuring the adaptation of cyanobacteria to environmental conditions. It is noteworthy that numerous studies have identified the potentials of cyanobacteria for modern drug discovery due to the bioactivities exhibited by cyanobacterial metabolites.

## 2. Adaptation Strategies of Cyanobacteria

### 2.1. Physiological Adaptation

Cyanobacteria possess the capacity to switch from one mode of metabolic approach to another. Most cyanobacteria conduct oxygenic photosynthetic mode. However, some can switch to anoxygenic photosynthetic mode [29]. For example, a filamentous mat of *Leptolyngbya* sp. and a cyanobacterial community of *Planktothrix* sp. and *Annamia* sp. dominating the sulfidic water column can conduct anoxygenic photosynthesis using sulfide as an electron donor [30,31]. Moreover, some cyanobacteria can carry out the fermentation processes under anoxic conditions and in the dark [32]. On the other hand, many cyanobacteria species form heterocysts, the cells that carry out atmospheric nitrogen fixation, especially during nitrogen deprivation [33]. This uniquely differentiated cell results in the dispersion of cyanobacterial genera in various ecosystems; for example, *Anabaena* and *Trichodesmium* inhabit open oceans, thermal springs and freshwaters [34,35], whereas *Leptolynbya* grows in geothermal springs, hot deserts, and surface crusts of semi-deserts [36,37].

The high adaptability of cyanobacteria to high temperature environments might be related to their photosynthetic machinery acclimation throughout many years of evolution. Previous studies have identified that light-harvesting phycobilisome (PBS) and photosystem II (PSII) are the main components that contribute to the survival of thermophilic cyanobacteria. *Synechococcuss* A/B clade, *Mastigocladus laminosus*, *Synechococcus lividus* and *Synechococcus vulcanus* have developed PBS with a greater thermostability during the evolutionary divergence [38]. The rigidity of the phycocyanin complex is important in achieving PBS thermostability [39]. On the other hand, D1 protein and PsbU, the key subunits of PSII, provide stability to PSII from denaturation at a high temperature [40,41]. Previous studies have also reported that filamentous cyanobacteria are thermostability related to metabolic mechanisms, which enables them to survive at high temperatures [42,43].

Additionally, some marine planktonic cyanobacteria, for example, *Synechococcus* sp. PCC 7942, exhibit DNA repair mechanisms, including detoxifying enzymes and pigments [44] and UV-absorbing sunscreen molecules [45] to release the damage caused by UV radiation and to protect them from harmful radiation pollutants [46]. Many planktonic cyanobacteria possess gas vesicles for position adjustment in the water column. Cyanobacteria use these gas-filled structures in connection to different environmental stimuli such as photic, gravitational, chemical and thermal to find a suitable niche [47].

### 2.2. Cellular Morphological Adaptation

Cyanobacteria exist in different morphological structures: unicellular in a single cell or colony with or without mucilaginous envelope, unbranched filamentous with single or multiple trichomes with or without sheath, and branched filamentous [48]. Furthermore, cyanobacteria have been subdivided into five subsections according to their morphological characteristics, subsection I (unicellular), subsection II (unicellular with baeocytes), subsection III (unbranched filamentous without heterocyst), subsection IV (false-branched or unbranched filamentous with heterocysts) and subsection V (branched filamentous with heterocysts) [49]. Although the forms are not habitat dependent, these physical characteristics might have contributed to cyanobacterial evolutionary adaptations. The earliest cyanobacterial genera are unicellular with sheath living in freshwater habitats [50]. The sheath enables benthic or sessile cyanobacteria such as *Gloeocapsa*, *Synechococcus*, *Prochlorococcus* and *Aphanothece* to form epilithic/endolithic biofilms in water bodies. This thick protective layer also provides high irradiance and UV light defense to the cells. Remarkably, some unicellular cyanobacteria possess thin firm colorless sheath for adaptations in extreme habitats, such as *Chroococcidiopsis*, which can be found in thermal and mineral springs, alkaline hypersaline swamps and hot or Antarctic deserts [51] as well as *Chroococcus*, which can be found in thermal springs and calcite speleotherms [52,53]. In contrast, the solitary cells or small groups of *Halothece* that inhabit coastal salty habitats lack mucilaginous envelope [54]. Noteworthy, most filamentous cyanobacteria produce extracellular sheath as a method of adaptation, especially to water level fluctuation and high solute concentration, by providing a microenvironment for trichomes. For example, *Microcoleus*, *Trichocoleus*, *Oscillatoria* and some *Schizothrix* covered by thick sheath grow in saline soil crusts, semi-desert regions, soil crusts of desert and polar environments [35,55,56]. However, some mat-forming *Schizothrix* and *Oscillatoria* enveloped by firm and thin sheaths inhabit diverse aquatic environments, freshwater, marine environments, thermal springs and polar water bodies [55]. Moreover, heterocystous cyanobacteria, such as *Anabaena* and *Trichormus*, have filaments without sheath or gelatinous envelope, which are necessary to allow more light penetration into the cells. In the case of cyanobacterial symbionts (cyanobionts), the absence of sheath or gelatinous envelope is important to enhance nitrogen and carbon transfer between the symbiotic partners [55]. 

### 2.3. Bioactive Metabolites for Cyanobacterial Adaptations and Their Pharmacological Properties

#### 2.3.1. Indole Alkaloids

Alkaloids are ubiquitous in plants, bacteria, fungi and animals. In plants, alkaloids are produced as secondary metabolites in response to biotic or abiotic stresses [57]. Indole alkaloids are one of the alkaloid classes consisting of one indole structural moiety and are known for their bioactivities [58]. Many studies were conducted on the pharmacological properties of indole alkaloids from plants, fungi and animals, for example, antitumor activities showed by vinblastine and vincristine from *Catharanthus roseus* [59] and UV protective function by pityriacitrin from yeast *Malassezia furfur* [60], as well as the anti-inflammatory effect produced by conicamin from tunicate [61], lepadiformines A and B from ascidian [62], manzamine and carteramine A from sponges [63,64], and ascidiathiazones A and B from ascidan [65,66].

A diverse class of indole alkaloids synthesized by cyanobacteria have been reported as the bioactive secondary metabolites that possess pharmacological and biological properties (Table 2). 

**Figure 1:**
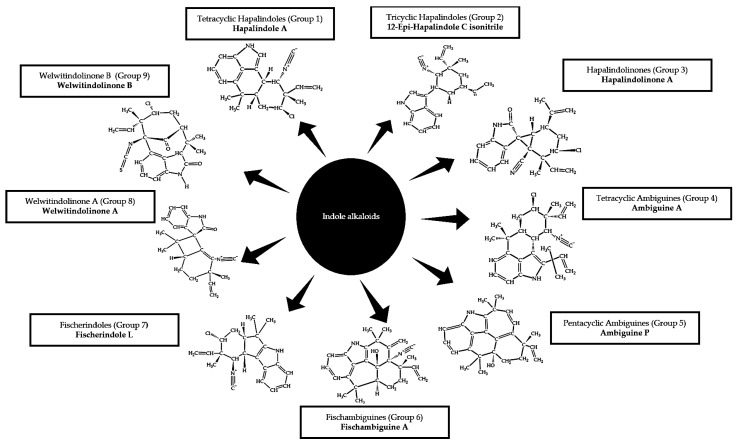
Classification of indole alkaloids from Stigonematales order of cyanobacteria [81].

Thus far, 80 variants of indole alkaloids have been identified exclusively produced by the genera *Westiella*, *Westiellopsis*, *Fischerella* and *Hapalosiphon* (belonging to subsection V formerly order Stigonematales) [79,80,81]. The variants belong to nine different groups based on their carbon skeletons (Figure 1). Hapalindoles are the largest group of alkaloid indoles produced by cyanobacteria, which make up Group 1 (tetracyclic hapalindoles) and Group 2 (tricyclic hapalindoles). Furthermore, the hapalindoles are the precursors for the other groups, so-called hapalindole-type alkaloids: the hapalindolinones (Group 3), the ambiguines (Group 4 and Group 5), fischambiguines (Group 6), fischerindoles (Group 7) and welwitindolinones (Group 8 and Group 9). Such an extensive list of indole alkaloids suggests the important roles of these secondary metabolites in ensuring the survival of the cyanobacterial genera. Although most of the indole alkaloids are identified from the terrestrial and freshwater cyanobacteria in the genera *Westiella*, *Westiellopsis*, *Fischerella* and *Hapalosiphon*, it is tempting to speculate that these secondary metabolites are also produced by these genera inhabiting the other ecosystems, especially by those thriving in the extreme environments. Remarkably, the hapalindole family appears to be inherited vertically and thus, suggests the inheritance of hapalindole biosynthetic genes within the Subsection V [73].

Interestingly, scytonemin, an indole alkaloid UV-filtering pigment, is predominantly produced by cyanobacteria [82,83]. A recent study reported that an unculturable *Halothece* produced scytonemin in response to UV-A radiation at the driest Salar Grande, Atacama Desert [84]. In addition, scytonemin found in the terrestrial *Lyngbya* sp. CU2555, showed high resistance toward UV-B and heat, thusprotecting the cells against harsh environmental conditions. This strong oxidizing agent not only functions as a photoprotective compound against harmful UV radiation but also provides protection against deleterious short-wavelength radiation [85]. Notably, the accumulation of scytonemin in the unicellular *Chroococcidiopsis*-like cyanobacterial isolate from an epilithic desert crust occurred due to the increase in both temperature and photooxidative conditions together with UV-A exposure. However, the increased salt concentration under UV-A radiance blocked the production of scytonemin [86].

Furthermore, β–carboline, another indole alkaloid compound that is widely distributed in plants, animals and human tissues [87], has also been detected in cyanobacteria. It is noteworthythat norharmane (9H-pyrido(3,4-b) indole) excreted by *Nodularia harveyana* exhibited high algicidal activity [88]. In addition, this indole alkaloid can highly inhibit Gram-positive bacteria and moderately control Gram-negative bacteria and yeast [89]. Moreover, nostocarboline from *Nostoc* 78–12A could be used as an acetylcholinesterase inhibitor for the treatment of neuronal diseases [90].

#### 2.3.2. Terpenoids

**Figure 2:**
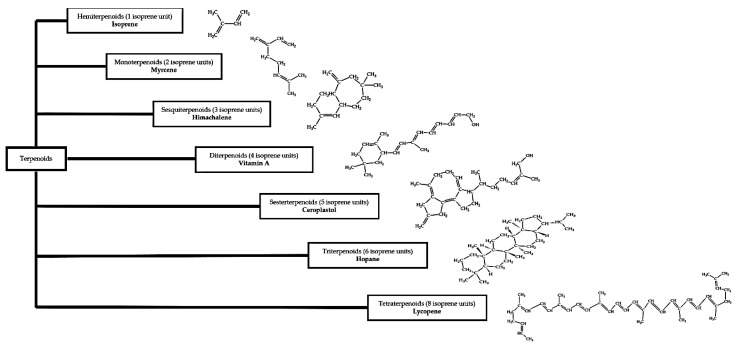
Classes of terpenoids based on their isoprene units [93].

Terpenoids (or isoprenoids) are the largest group of bioactive compounds with more than 55,000 compounds discovered to date [91]. They can be classified as hemiterpenoid, monoterpenoids, sesquiterpenoids, diterpenoids, sesterterpenoids, triterpenoids (steroids) and tetraterpenoids (carotenoids) based on the number of isoprene units (Figure 2) [92].

In plants, terpenoids are involved in primary growth and development, defense against predators, and endophytic fungi or bacteria, as well as the attraction of pollinators [94]. These odorous metabolites are also synthesized by many bacteria including cyanobacteria that produce earthy odors in soil and water resources [95,96]. A range of terpenoids havebeen found in cyanobacteria and are known for their essential roles in ensuring cyanobacterial survival in a vast environment, as well as their importance as medicines, pigments and flavors.

Terpenoids identified from the halophilic *Cylindrospermum muscicola* HPUSD12 and *Phormidium* sp. HPUSD13, are suggested to play roles in providing protection against free radical oxidative damage to the cells that might be caused by the high-salinity condition of the Drang salt mine in India [97].

Sesquiterpenoid geosmin, a sesquiterpene without an isopropyl group, can be produced by several freshwater cyanobacteria, such as the filamentous *Calothrix* PCC 7507 [98] and the unicellular *Synechocystis* sp. PCC 6803 [99]. Furthermore, the heterologous expression of the sesquiterpene synthase gene from *Nostoc punctiforme* PCC 73102 and *Nostoc* sp. PCC 7120 in *Escherichia coli* suggests the production of sesquiterpenoids by this versatile species that inhabits various aquatic and terrestrial ecosystems [100]. This terpenoid group regulates the signaling defense activities of the cyanobacteria in response to the environmental stimuli. Interestingly, sesquiterpenoids produced by the marine *Oscillatoria spongeliae* are suggested to be responsible for the symbiotic interaction with the tropical marine sponge [101].

Triterpenoids, such as 2-methylhopanoids (2-MeBHPs), were discovered in a significant quantity in both laboratory cyanobacterial cultures and natural cyanobacterium-dominated microbial mats [102,103]. 2-MeBHPs is an example of pentacyclic triterpenoids, which play the role of biomarkers for modern cyanobacteria in some environmental settings [103]. Moreover, 2-MeBHP promotes osmotic, pH stress and freezing/thawing resistance in cyanobacteria to ensure their survival in dessert soil crusts, hot springs, hypersaline lake, Antarctic water, and Artic soil [100]. Apparently, the deletion of *hpnP*, the gene coding for the hopanoids protein responsible in C-2 methylation, caused a decrease in osmotic and pH stress tolerance by *Nostoc punctiforme* ATCC 29133S [104].

Tetraterpenoids, also known as carotenoids, are ubiquitousin most photosynthetic organisms and are essential for light-harvesting and energy dissipation during the photosynthesis process [105,106]. β-carotene, zeaxanthin, and echinenone are common carotenoids produced by cyanobacteria. The freshwater *Aphanothece microscopica Nägeli* (RSMan92) [107] and both the marine *Cyanobium* sp. LEGE 06113 [108] and *Trichodesmium* sp. [109] are also excellent sources of these terpenes. Carotenoids are lipophilic secondary metabolites from the isoprenoid pathway that are necessary to facilitate cyanobacteria against direct UV light exposure and photooxidative damage while conducting photosynthesis. In particular, it was suggested that echinenone and zeaxanthin protect PSII against singlet oxygen [110]. In addition, *Pseudanabaena* sp. CCNU1, inhabiting the arid and exposed region in Chetimari, Niger, produces carotenoids, specifically, β-carotene, echinenone, canthaxanthin, zeaxanthin, synechoxanthin, and three myxoxanthophyll derivatives, as a method of protection against UV-B radiation [111]. Moreover, carotenoids also play important roles in the survival of endolithic cyanobacteria, *Chroococcidiopsis* sp. at the Atacama Desert [112].

Recent studies have reported the biological activities of cyanobacterial carotenoids for the treatmentof various diseases. Several cyanobacteria strains, including the freshwater *Alkalinema aff. pantanalense* LEGE15481, *Cyanobium gracile* LEGE12431, *Cuspidothrix issatschenkoi* LEGE03282, the terrestrial *Nodosilinea* (*Leptolyngbya*) *antarctica* LEGE13457 and the marine *Leptolyngbya*-like sp. LEGE13412, have been characterized for their high content of carotenoids [113]. Remarkably, carotenoids and their derivatives extracted from terrestrial and marine cyanobacteria showed high superoxide anion radical (O_2_^•−^) scavenging and anti-inflammatory effects that enabled the treatment of psoriasis [113]. A high number of carotenoids was also detected in *Cyanobium* sp. LEGE 07175 and *Tychonema* sp. LEGE 07175 [114]. Both cyanobacterial extracts showed strong antiaging effects by inhibiting hyaluronidase, the enzyme that stimulates the depolymerization of hyaluronic acid under oxidative stress [114]. Although the information on the biochemical mechanisms of carotenoids in cell apoptosis and proliferation is still scarce, their antioxidantcapacity might be a contributing factor in anticancer and anti-aging effects.

Moreover, scytoscalarol (also known as an antimicrobial sesterterpene) from *Scytonema* sp. (UTEX 1163) culture showed growth inhibition against *Bacillus anthracis*, *Staphylococcus aureus*, *Escherichia coli*, *Candida albicans*, and *Mycobacterium tuberculosis* [115]. In addition, cybastacines A and B found in *Nostoc* sp. BEA-0956 also showed antibacterial activities against some clinical pathogenic bacteria [116]. Remarkably, scytonemides A and B extracted from the freshwater *Scytonema hofmannii* (UTEX 1834) havebeen identified and can function as an anticancer agent through the inhibition of 20S proteasome, the catalytic core of the proteasome complex that catalyzes the degradation of regulatory proteins [117].

#### 2.3.3. Mycosporine-Like Amino Acids (MAAs)

Mycosporine-like amino acids (MAAs) are UV-absorbing compounds are involved in the evolution of organisms living in environments with high exposure to sunlight, such as cyanobacteria, microalgae, fungi, seaweeds, corals, and lichens [118,119,120,121]. These small water-soluble compounds (generally <400 Da) provide protection against ultraviolet radiation (UVR) exposure. In fact, the production of MAAs is induced by UV radiation and osmotic stresses; however, the mechanism remains poorly understood [122]. On the other hand, these photoprotective compounds also exhibit antioxidant activity [123,124,125].

*Synechococcales*, *Chroococcales*, *Oscillatoriales*, and *Nostocales* are efficient producers of MAAs for adaptations [118,126]. For example, a study found that MAAs produced by the benthic filamentous cyanobacteria in the Alpine Lake Gossenköllesee function as a protective shield against UVR. Despite a very low turnover of the synthesis of MAAs by the benthic filamentous cyanobacteria, especially during the ice-free season, these secondary metabolites reduce the transmission of UVR wavelengths received by the cyanobacteria in the clear Alpine lakes [127]. MAAs also provide protection against the damaging effects of solar UVR to the cyanobacterial mats in the Arctic [128]. Moreover, the MAA *mys* gene cluster in the filamentous *Chlorogloeopsis fritschii* PCC 6912 was up-regulated when simultaneously exposed to both UV and far-red lights. Thissuggests that MAAs may be involved in photon dissipation and thermodynamic optimization, which are important in regulating the heat from affecting the climate [129,130].

Recently, an usual mycosporine-glycine-alanine (MGA), an MAA derivative, was found in *Sphaerospermopsis torques-reginae* ITEP-024 [123]. Inhabiting freshwater with low salinity, this heterocystous filamentous cyanobacterium also produces the imino-mycosporines, shinorine and porphyra-334, as an acclamatory response to UV exposure [123]. Other than providing protection against UV light, rear mycosprorine-2-glycine (M2G), isolated from the halotolerant *Aphanothece halophytica*, possesses biological functions, such as free radical scavenging [131], oxidative stress protection [132], and osmoregulation [133], as well as inhibition of collagenase activity and protein glycation [134].

Interestingly, MAAs are attractive to cosmetic industries as the active ingredients for sunscreens and anti-aging products due to their characteristics [119,135]. For example, a recent in vitro study identified the protection activity of human keratinocytes against UV radiation by a novel MAA (13-O-β-galactosyl-porphyra-334) from *Nostoc sphaericum* [136]. This novel MAA also possesses radical scavenging activity that can reduce the damage caused by ROS in order to prevent photoaging. Nevertheless, MAAs have yet to be exploited for industrial production, with only a few edible products currently available. For example, both Helioguard 365® and Helionori® extracted from the red seaweed *Porphyra umbilicalis* [137,138] have been used as ingredients for the production of sunscreens. The in vivo study using Helioguard 365® from the red seaweed showed improvements in skin firmness and smoothness [138], whereas Helionori® offered protection against DNA damage due to UV radiation [139]. Nevertheless, these products are able to provide maximal protection in the UVA range but only allow minimum protection in the more damaging UVB range [125].

#### 2.3.4. Non-Ribosomal Peptides and Polyketides

Biosynthesis of non-ribosomal peptides (NRPs) and polyketides (PKs) are catalyzed by non-ribosomal peptide synthases (NRPS) and polyketide synthases (PKS), respectively [140]. Notably, the gene clusters of NRPS and PKS were more frequently found in bacteria, including cyanobacteria, than archaea and eukarya [141]. The gene clusters have been found in the genera *Lyngbya*, *Microcystis*, *Planktothrix*, *Nodularia*, *Nostoc*, *Pleurocapsa*, and *Anabaena* [141,142]. *Pleurocapsa* and *Nostoc* species are the most common cyanobacteria producing NRP/PK [141]. However, cyanobacteria with genomes fewer than 3 Mbp, such as *Prochlorococcus marinus* SS120 and *Synechococcus* sp. WH8109, might not possess these clusters due to the extra metabolic burdens [141,143,144].

The biosynthesis of peptides and polyketides in microorganisms is a unique modular pathway regulated by NRPS and PKS. NRPS comprises modules, each of which integrates proteinogenic amino acids with non-proteinogenic amino acids, fatty acids, carbohydrates and other building blocks into peptide chains [142]. These peptide-synthesizing enzymes accept approximately 300 proteinogenic and nonproteinogenic substrates during the biosynthesis of non-ribosomal peptides [145]. In bacteria, PKS Type I are widely found to be responsible for polyketide chain elongation, processing and termination [146]. These modular enzymes are involved in the recognition, activation and condensation of coenzyme A (CoA) derivatives as the building blocks [141,146]. Not only are the secondary metabolites produced through this unique natural combinatorial biosynthetic pathway are important for growth, symbiotic interactions and protection against biotic and abiotic stresses, but they also possess various therapeutic activities (Table 3). Some of the compounds have been applied for the treatment of various acute and chronic diseases.

#### 2.3.5. Ribosomal Peptides

Ribosomal peptides (RPs) are peptide chains of proteinogenic amino acids that can be found on ribosomes. In contrast to the biosynthesis of NRPs, only 20 proteinogenic amino acids are used as the building blocks during the biosynthesis of RPs [145]. There are three major RP families: cyanobactin, microviridins, and lantipeptides. Cyanobactin is diversely present in symbiotic and planktonic cyanobacteria [166]. Microviridins are the largest RPs, consisting of between 12 and 20 amino acids, which have been classified into four classes (Group I–IV) and are found in freshwater cyanobacteria [167]. Lantipeptides can be produced by four different classes (Class I–IV) of lantipeptidase in the cytosol of producing strains. Few studies have been conducted on cyanobacteria producing lantipeptides. However, comparative genomic analyses revealed that the marine *Prochlorococcus* and *Synechococcus* possess Class II lentipeptidase, ProcM [168,169]. The heterologous expression of *procM* and *procA* genes identified that ProcM can catalyze the dehydration and cyclization of all 29 different ProcA precursor peptides to produce the lantipeptides called prochlorosins [170]. Such an efficient biosynthetic pathway for generating prochlorosins with structural diversity is necessary for *Prochlorococcus* strains MIT9313 and MIT9303, as well as *Synechococcus* strain RS9916, which has a small genome size. Remarkably, RPs exhibit biological activities that could be used as natural drugs in the future (Table 4).

It is unclear on how the NRPs, PKs and RPs are involved in the survival of cyanobacteria; however, these posttranslational modified compounds are well known for their antibacterial properties, which could be important when in competition with other microbial species in the ecosystem [170]. For example, under poor nutrient conditions in hot springs, cyanobacteria and other bacterial classes, such as Deinococci, Alphaproteobacteria, Ignavibacteria, and Betaproteobacteria [180], may competefor organic and non-organic matters, as well as space for either exposure to sunlight or cover from direct sunlight. Additionally, benthic or sessile cyanobacteria may produce NRPs, PKs, and RPs for cell signaling in order to form epilithic/endolithic biofilms in water bodies, saline soil crusts or soil crusts of desert and polar environments. Moreover, the oligopeptides produced by cyanobacteria could be crucial for other organisms, such as eukaryotic algae, sponges, and plants, used as precursors for their metabolic pathways.

#### 2.3.6. Phenolic Acids

Phenolic acids consist of one carboxyl group and one or more hydroxyl groups joined to the aromatic ring. These secondary metabolites are one of the largest groups of phenolic compounds. Phenolic acids are represented by hydrocinnamic acid, hydrobenzoic acid, phenylacetic acid and phenylpropionic acid derivatives from the shikimate pathway [181,182]. Phenolic acids produced by photosynthetic organisms are necessary for protection against oxidative damage that might be caused by reactive oxygen species (ROS) and the hydroxyl radical (OH).

In cyanobacteria, the accumulation of phenolic acids ensures the tolerance and adaptability of these photosynthetic microbes to various environmental stresses, which can cause the deposit of free radicals in cells, as well as chemical damage to deoxyribose and DNA. Notably, the accumulation of gallic acid, caffeic acid, chlorogenic, ferulic acid, and vanillic acid was detected when a high concentration of NaCI was supplied into the cultures of *Plectonema boryanum*, *Haplosiphon intricatus*, *Anabaena doliolum,* and *Oscillatoria acuta* [183], and thus suggesting that these phenolic acids play roles in the scavenging of free radicals under salt stress conditions. A recent study reported that the abundance of phenolic compounds in response to both cold and hot shocks might have stimulated the antioxidant capacity in halotolerant *Halothece* sp. PCC 7418 [184]. It is noteworthythat the synergistic effect of phenolic acids and other antioxidative compounds (flavonoids, MAAs and phycobiliproteins) is necessary for the response.

As with other phenolic compounds, many studies identified that plant phenolic acids also manifest antimicrobial [185] and antiviral properties [186]. Due to their ability to reduce oxidative damage or stress in cells, phenolic acids such as gentisic acid, gallic acid and syringic acid exhibitgood recovery in heart failure [187], memory loss [188] and wound healing [189], respectively. Previous studies have also reported that ferulic acids can produce skin whitening and anti-wrinkle effects [190] whereas gallic acid has anti-aging properties [191]. These therapeutic effects might also be produced by cyanobacterial phenolic acids due to their abilities to detoxify ROS and scavenge free radicals [183,184].

#### 2.3.7. Flavonoids

Flavonoids are polyphenolic compounds that are widely distributed in plants [192]. These secondary metabolites can be classified into different subclasses: chalcones, flavanols, flavanones, flavones, isoflavones, flavonols, and anthocyanins. Remarkably, plant flavonoids exhibit antioxidant, anticancer, antiviral and anti-inflammatory properties [193,194].

In addition to phenolic acids, flavonoids are also antioxidants that are important for the survival of cyanobacteria. These antioxidative molecules, particularly quercertin and lutin, might facilitate *Plectonema boryanum*, *Haplosiphon intricatus*, *Anabaena doliolum*, and *Oscillatoria acuta* in salt acclimation mechanisms [183]. On the other hand, the chromatographic analysis identified that the thermophilic *Leptolyngbyba* sp. produces a high amount of luteolin-7-glucoside and naringenin [195], which could protect the cells from oxidative damage due to high temperatures. Additionally, naringenin not only plays a role as a strong free radical scavenger but also affects the growth and physiological functions of the halophilic *Spirulina platensis* and *Arthrospira maxima* and the freshwater *Anabaena* sp. by altering the cell wall and cellular membrane permeability [196]. These features are crucial to allow the secretion of exopolysaccharides (EPS) onto the surface of cyanobacterial cells for protection against unfavorable environmental conditions [196]. The oxidative power of the total flavonoids produced by cyanobacterial strains [183,184,195,197,198] suggests that these strong antioxidative compounds might also have pharmacological potentials similar to the plants flavonoids, such as nephroprotective [199], neuroprotective [200], anticancer [201], and antiatherosclerotic properties [202,203].

#### 2.3.8. Vitamins

Vitamins are commonly synthesized by photosynthetic organisms. Vitamin B: B1 (thiamin), B2 (riboflavin), B3 (niacin), B5 (pantothenic acid), B6 (pyridoxine), B7 (biotin), B9 (folic acid), B12 (cobalamin), and C are water-soluble compounds, whereas vitamin A, D, E, and K are lipid-soluble compounds. Plants produce vitamin A, B, C, E, and K in most organ parts to alleviate the effects of environmental stresses [204]. However, not all plants produce all vitamins and in fact, vitamin D and K, as well as some types of vitamin B are scarcely present [205]. By contrast, microalgae including cyanobacteria can produce vitamin D, K and B12, which are not present in higher plants [205].

*Arthrospira maxima*, *Anabaena cylindrica*, and *Synechococcus* sp. displayed high contents of β-carotene (pro-vitamin A) [206,207]. Remarkably, these cyanobacteria produce much higher β-carotene than some fruits, such as carrots and oranges [205]. Similarly to in plants, this pro-vitamin A carotenoid compound produced by the cyanobacteria possesses a great oxidative efficacy against ROS, which is important for photooxidative protection. It is noteworthy that the efficacy of O^2•−^ scavenging by β-carotene is greater than vitamin E and C [205].

Cyanobacteria are the major sources of B vitamins in marine and freshwater ecosystems. These water-soluble vitamins secreted by some cyanobacteria into the water bodies are important nutrients for other aquatic organisms [206,208,209]. Additionally, B vitamins might also be necessary in the metabolic pathways of cyanobacteria [205], as a high content of B2, B5 and B6 was detected in the freshwater *Anabaena cylindrica* [206] whereas, a marine *Anabaena cylindrica* was found to produce a high amount of B12. On the other hand, chromatographic analysis detected a high content of B2, B3, B9 and B12 in dried biomass of commercial *Arthrospira maxima* and *Arthrospira platensis* [210]. Notably, vitamin B also plays a role in cyanobacterial adaptation to environmental stresses. *Nodularia spumegina* accumulates B1 in response to salinity and temperature stresses [208]. Together with β-carotene, B1 ensures that this planktonic cyanobacterium is able to survive under high UV radiation [208].

Other than the higher plants, *Anabaena cylindrica* is also an excellent source of vitamin C [206]. This well-known antioxidant compound may provide protection against oxidative compounds in cyanobacteria.

A very low amount of vitamin D was detected in *Arthrospira* sp. [211]. As characterized in other algae species, this lipid-soluble vitamin might be important to ease the damage or degradation of cell membranes in cyanobacteria caused by UV radiation.

A high amount of vitamin E has been found in *Nostoc* sp. PCC 7120, *Synechocystis* sp. PCC 6803, *Anabaena cylindrica*, *Synechococcus* sp. PCC 7942 and *Arthrospira maxima* [206,207,212,213]. Remarkably, the vitamin E content is higher in these cyanobacteria compared to some common food sources [206]. The production of vitamin E is necessary for protection against photooxidative damage to PSII [214]. The accumulation of α-tocopherol was stimulated by the light intensity when *Synechocystis* sp. PCC 6803 was grown under photoautotrophic conditions [212]. Additionally, vitamin E also facilitates cyanobacteria to survive an emerging nutrient limitation. A study showed that *Arthrospira maxima*, *Nostoc* sp. PCC 7120 and *Synechocystis* sp. PCC 6803 produce low amount of vitamin E under optimum nitrogen availability [213]. However, *Arthrospira* sp. and *Oscillatoria* sp. synthesize high amounts of vitamin E in response to nitrogen deficiency at their logarithmic growth phase. Moreover, it is known that microalgae also produce vitamin E in response to nutrient limitation [215]. It is noteworthy that the production of vitamin E in microalgae is also a reaction in response to oxidative stress caused by metals [216,217]. This same antioxidative response could also happen in cyanobacteria, although so far, no study has been reported in these photosynthetic bacteria.

It was proposed that the marine *Anabaena cylindrica* possess a higher content of vitamin K1 than spinach and parsley [218]. Conversely, *Spirulina* sp. CS-785 produces a low amount of K1. Phylloquinone (vitamin K1) is synthesized by most cyanobacteria such as *Anabaena variabilis*, *Mastigocladus laminosus*, *Nostoc muscorum*, *Prochlorococcus* sp., *Anacystis nidulans* and *Synechocystis* sp. PCC 6803 [219,220,221]. Phylloquinone not only acts as an one-electron carrier at the A1 binding site of PSI, but it also provides protection against growth damage at high light intensity [222]. Similar to vitamin K1, menaquinone (vitamin K2) acts as a secondary electron acceptor of PS1 in *Gloeobacter violaceus* and *Synechococcus* sp. PCC 7002 [223,224]. Although phylloquinone and menaquinone exhibit structural similarity, the latter compound was absent in *Synechococcus* sp. PCC 7002, and two enzymes involved in its biosynthesis were missing in *Gloeobacter violaceus*.

Humans obtain vitamins through their diet. Vegetables, fruit, fish, and meat are great sources of vitamins. Currently, vitamin deficiencies occuring in humans are treated with synthetic vitamin analogs. For example, intramuscular injections or oral vitamin B_12_ therapy are the most common treatments for patients with vitamin B_12_ deficiency [225]. Those with vitamin D deficiency can be treated with oral ergocalciferol (vitamin D_2_) [226]. For adults with vitamin C deficiency and scurvy signs, oral ascorbic acid followed by a nutritious diet are always recommended [227,228]. Moreover, vitamin K1 can be administered via intramuscular injection or orally for people with vitamin K deficiency [229]. For adults with deficiency of vitamin A, vitamin A palmitate in oil is the most common method of treatment [230,231]. 

To date, only *Spirulina* (*Arthrospira* sp.) has been made available for consumption by humans as a supplementary diet. Indeed, several in vivo studies have showed the health benefits of well-characterized *Spirulina*, a rich source of vitamin B12, β-carotene and vitamin E. For example, *Spirulina* can improve bone strength and stiffness due to vitamin B12 deficiency [232], prevent ulcer formation [233] and recover blood retinol status [234].

#### 2.3.9. Antimetabolites

Antimetabolites are small molecules that inhibit the biosynthetic pathway by binding to the active site of the target molecule. An unusual deoxy sugar, 7-deoxy-D-altro-2-heptulose (7-deoxysedoheptulose, 7dSh) obtained from the *Synechococcus elongatus* PCC 7942 culture supernatant was identified to show biological activity against several wild type organisms, specifically, *Anabaena variabilis*, *Saccharomyces cerevisiae* and *Arabidopsis thaliana* [235]. In vitro analysis suggested that this antimetabolite mimics 3-deoxy-D-arabino-heptulosonate 7-phosphate (DAHP), the substrate of 3-dehydroquinate (DHQ) synthase. The binding of 7dSH on DHQ synthase leads to the inhibition of the enzyme and consequently blocksthe shikimate pathway [235]. Additionally, a recent study detected the accumulation of 5-deoxyribose (5dR) and then 7dSh in the *Synechococcus elongatus* PCC 7942 culture supernatant under elevated CO_2_ conditions [236]. The formation of 5dR was reported to be derived from the 5-deoxyadenosine (5dAdo) salvage pathway as a detoxification strategy in order to protect the radical S-adenosyl-l-methionine (SAM) enzymes from feedback inhibition [236]. In fact, 5dR is continuously imported and exported by the cells and serves as a precursor for 7dSH, which is metabolized by transketolase activity when a relatively high extracellular 5dR concentration is reached [236]. This unique biosynthesis pathway strategy enables cyanobacteria to survive a niche competition by inhibiting the growth of other microalgae or bacteriaand is especially crucial for the unicellular cyanobacteria with small genome sizes and fewer plasmids [236].

Another cyanobacterial antimetabolite, a nonprotein amino acid β-methylamino-L-alanine (BMAA), may be involved in the nitrogen metabolism of cyanobacteria in order for the nitrogen-fixing microbes to survive under nutrient deprivation. Previous studies have suggested that the production of BMAA is correlated with nitrogen starvation under both natural and culture conditions, which results in the inhibition of the nitrogen assimilation pathway [237,238,239,240]. In turn, the concentration of BMAA was declined when a nitrogen source was added to the nitrogen-starved *Microcystis* PCC 7806 culture [241]. Although very little is known regarding the biological function of BMAA in cyanobacteria, *Synechococcus* sp. TAU-MAC 0499, *Synechocystis* PCC 6803 and *Anabaena* sp. PCC 7120 have been found to rapidly import exogenous BMAA [237,238,240]. BMAA was suggested to impair the activity of glutamine synthetase-glutamine-oxoglutarate aminotransferase (GS-GOGAT), the sequentially functioning enzymes that are involved in nitrogen assimilation, in the non-BMAA producer *Synechococcus* sp. TAU-MAC 0499 and the BMAA producer *Synechocystis* PCC 6803 [237,242]. Specifically, BMAA competes with glutamine to bind to GOGAT and acts as an inactivating factor [242]. On the other hand, nitrogenase activity was inhibited in *Anabaena* sp. PCC 7120 culture supplied with exogenous BMAA and the cyanobacterial growth was retarded by forming chlorotic cells. Similarly, the growth of BMAA producer *Synechocystis* PCC 6803 was arrested and the formation of chlorotic cells increased in the presence of exogenous BMAA [238]. Chlorotic is a dormant state of cyanobacterial cells in order to prolong their survival period under nitrogen starvation [243]. On the contrary, exogenous BMAA does not affect the physiology of *Synechococcus* sp. TAU-MAC 0499 [237]. A recent study reported a different response of growth retardation by the non-BMAA producers *Microcystis aeruginosa* (FACHB-836 and 905) [244] compared to *Synechococcus* sp. TAU-MAC 0499, whereas BMAA has no negative impacts on the BMAA producers *Anabaena* sp. FACHB-418 and *Microcystis wesenbergii* FACHB-908 [244]. To date, little is known about the effects of BMAA on cyanobacteria. However, it is tempting to speculate that cyanobacteria synthesize BMAA as a response to nutrient-limited conditions by either eliminating the competitors or forming dormant cells [237,238,244,245]. Additionally, the fact that a low concentration of bound BMAA was detected in the non-nitrogen-fixing *Microcystis wesenbergii* (FACHB-908) and Synechocystis (FACHB-898) suggested that BMAA may be involved in the formation of cyanobacterial proteins [244].

Remarkably, antibacterial, antifungal, and herbicidal properties exhibited by the unusual deoxy sugar 7dSh [235] suggest its applications in various fields including agriculture, water management, veterinary medicine, and human medicine. On the other hand, further confirmation on the biological function of BMAA in cyanobacteria could provide solutions to control cyanobacterial bloom and subsequently overcome its neurotoxic effects on humans, which are associated with amyotrophic lateral sclerosis, Parkinson’s disease, and Alzheimer’s disease [246].

## 3. Conclusions

Pharmacological effects exhibited by plant natural bioactive metabolites have led to their numerous applications in the treatment of serious and chronic diseases. In plants, bioactive metabolites are typically produced in low amounts as secondary metabolites. Therefore, a large amount of plant resources is required for traditional extraction methods of these compounds to obtain the industrial yield. However, these methods are known to be unsustainable. For decades, important discoveries of the biological activities possessed by the bioactive metabolites produced by cyanobacteria has attracted attention for modern therapy. These oxygenic photosynthetic microbes produce bioactive metabolites as a response to environmental stresses. Some of the compounds are produced in abundance to release the stress effects. Moreover, the emergence of synthetic biology tools has allowed the combinatorial synthesis of plant-derived biosynthetic genes involved in metabolic pathways to be heterologously expressed in cyanobacteria. In fact, the capacity to express P450, an enzyme involved in secondary metabolite production in plants, is beneficial in themetabolic engineering of cyanobacteria for the heterologous expression of the plant bioactive metabolic pathway. Together with this synthetic biology approach, the advancement in bioprocess engineering can produce natural bioactive compounds using sustainable approaches in order to meet industrial demands in the future.

## Figures and Tables

**Table 1:** Different classes of cyanobacterial species based on their physiological characteristics.

Class	Habitats	Cyanobacteria	References
Alkaliphiles	Hypersaline swamps, alkaline-saline lake or ponds, hot spring, alkaline hot spring, alkaline-saline volcanic lake, soda deserts	*Microcoleus* sp., *Pleurocapsa* sp., *Synechococcus* sp., *Cyanobacterium* sp., *Spirulina subsalsa, Spirulina platensis, Spirulina maxima,* and *Arthrospira* sp.	[1,2,3,4,5,6,7,8,9,10]
Acidophiles	Sulfuric pools and acid mine drainage	Cyanobacteria cannot survive under this condition.	[11,12,13]
Endolithic	Rocks, granites and quartzites in desert, freshwater	*Chroococcidiopsis*-like cyanobacterium	[14]
Halophilic	Hypersaline lakes, coastal hypersaline lagoons, saline springs, salt flats and ponds	*Synechococcus* sp., *Leptolyngbya* sp, *Nodosilinea* sp., and *Geitlerinema* sp	[15]
Oligotrophics	Coastal regions of marine and freshwater	*Dolichospermum lemmermanii*	[16,17]
Psychrophilic	Alpines and polar regions	*Nostoc* sp., *Leptolyngba* sp., *Oscillatoria* sp. and *Phormidium* sp.	[18,19,20]
Thermophilic	Thermal springs and soil crusts of deserted area	*Synechococcus* sp., *Thermosynechococcus vulcanus, Leptolyngbya* sp., *Thermosynechococcus elongatus,* and *Phormidium* sp.	[21,22,23,24,25]
Radiophiles	Marine, freshwater and desert	*Synechocystis* sp., *Chroococcus minutus, Leptolyngbya* sp., *Trichodesmium* and *Crocosphaera*	[26,27,28]

**Table 2:** List of cyanobacteria producing diverse class of indole alkaloids.

Cyanobacteria species	Habitat	Compounds	Bioactivities	References
*Hapalosiphon* sp. CBT1235	Terrestrial	Hapalindoles	Inhibit T Cell Proliferation	[67]
*Hapalosiphon fontinalis*	Soil	Hapalindoles	Antibacterial and antimycotic	[68]
*Hapalosiphon fontinalis*	Soil	Hapalindoles	Antialgal	[69]
*Westiellopsis* sp. (SAG 20.93) and *Fischerella muscicola* (UTEX LB1829)	Freshwater and terrestrial	Hapalindoles	Antibacterial	[70]
*Fischerella ambigua* UTEX1903	Terrestrial	Ambiguine	Unknown	[71]
*Hapalosiphon welwitschii* UTEX B1830	Freshwater	Welwitindolinone	Unknown	[72]
*Westiella intricata* UH strain HT-29-1	Freshwater	Welwitindolinone	Unknown	[73]
*Fischerella ambigua* (UTEX 1903)*,Westiellopsis prolifica* and *Hapalosiphon hibernicus* BZ-3-1	Terrestrial	Ambiguine Isonitriles	Fungicidal	[74]
*Fischerella muscicola*	Terrestrial	Fischerindole	Antifungal	[75]
*Fischerella ambigua* (UTEX 1903)	Terrestrial	Fischambiguines and ambiguines	Antibacterial	[76]
*Fischerella* sp.	Terrestrial	Welwitindolinones	Multi-drug resistance reversing activity	[77]
*Hapalosiphon welwitschia* and *Westiella intricata*	Soil	Welwitindolinones	Multi-drug resistance re versing activity and insecticidal activity	[78]

**Table 3:** Bioactivities of non-ribosomal peptides (NRPS) and polyketides (PKS) produced by cyanobacteria.

Cyanobacteria species	Habitat	Compounds	Bioactivities	References
*Microcystis aeruginosa*	Freshwater	Microcystins	Inhibit eukaryotic types 1 and 2A phosphatases, cytoskeletal collapse, massive hepatic bleeding, potential tumor promoters and carcinogens	[147,148,149]
*Planktothrix agardhii* NIVA-CYA 126	Freshwater	Aeruginosin	Inhibit serine proteases	[150]
*Cylindrospermopsis raciborskii*, *Aphanizomenon ovalisporum* and *Aphanizomenon flos-aquae*	Freshwater	Cylindrospermopsin	Cytotoxic, neurotoxic effects and carcinogen	[151]
*Anabaena* sp. 90	Freshwater	Anabaenopeptin	Inhibit proteases	[152]
*Lyngbya bouillonii*	Marine	Apratoxin	Reversible inhibition of several cancer-associated receptors	[153,154]
*Lyngbya majuscula*	Marine	Lyngbyatoxin	Potent skin irritant	[155]
*Lyngbya majuscule* JHB	Marine	Hectochlorin	Antifungal and anticancer activity	[156]
*Lyngbya majuscule* 19L	Marine	Barbamide	Anti-molluscidal	[157]
*Lyngbya majuscule* 19L	Marine	Curacin A	Antiproliferative and cytotoxic activities	[158]
*Lyngbya majuscule* JHB	Marine	Jamaicamide	Block sodium-channel	[159]
*Nostoc* sp. GSV 224	Terrestrial	Nostopeptolide	No cytotoxic, antifungal and inhibit protease activities	[160]
*Nostoc* sp. ATCC 53789	Terrestrial	Nostocyclopeptide	Antitoxin activity	[161]
*Nostoc* sp. ATCC 53789	Terrestrial	Cryptophycins	Tubulin-destabilizing compound	[162]
*Cylindrospermum alatosporum* CCALA 988	Terrestrial	Puwainaphycins	Cytotoxic	[163]
*Nostoc calcicola*	Wastewater	Nostophycin	Antibacterial and antifungal	[164]
*Nodularia spumigena* NSOR10	Freshwater	Nodularin	Inhibits phosphatase type 1 and 2A, cytoskeletal collapse, massive hepatic bleeding, potential tumor promoters and carcinogens	[165]

**Table 4:** Bioactivities of ribosomal peptides (RPs) produced by cyanobacteria.

Cyanobacteria Species	Habitat	Compounds	Bioactivities	References
Cyanobactin				
*Microcystis aeruginosa*	Freshwater	Aerucyclamide A, B, C and D	Cytotoxic and antimalarial	[171,172,173]
*Stigonema dendroideum*	Terrestrial	Dendroamide A	Multidrug-resistance reversing activity	[174]
*Trichodesmium erythraeum*	Marine	Trichamide	No biological effects found	[175]
*Prochloron didemnid* (symbioant)	Marine	Patellamide A and C	Cytotoxic	[176]
*Anabaena* sp. 90	Freshwater	Anacyclamide	Cytotoxic	[177]
*Microcystis aeruginosa* PCC 7806	Freshwater	Microcyclamide	No biological effects found	[171]
Microviridin				
*Microcystis aeruginosa* NIES-298	Freshwater	Microviridios B and C	Inhibits elastase	[178]
Lantipeptides				
*Prochlorococcus* MIT9313	Marine	Prochlorosins	Bacteriocidal and act as signaling molecules	[168,170,179]

## Data Availability

Not applicable.
